# Causal information changes how we reason: a mixed-methods analysis of decision-making with causal information

**DOI:** 10.3389/fcogn.2025.1608842

**Published:** 2025-08-11

**Authors:** Samantha Kleinberg, Cristina Leone, Alice Liefgreen, David A. Lagnado

**Affiliations:** ^1^Computer Science Department, Stevens Institute of Technology, Hoboken, NJ, United States; ^2^Experimental Psychology, Division of Psychology and Language Sciences, University College London, London, United Kingdom

**Keywords:** causal models, decision-making, knowledge, beliefs, mixed-methods

## Abstract

Causal information, from health guidance on diets that prevent disease to financial advice for growing savings, is everywhere. Psychological research has shown that people can readily use causal information to make decisions and choose interventions. However, this work has mainly focused on novel systems rather than everyday domains, such as health and finance. Recent research suggests that in familiar scenarios, causal information can lead to worse decisions than having no information at all, but the mechanism behind this effect is not yet known. We aimed to address this by studying whether people reason differently when they receive causal information and whether the type of reasoning affects decision quality. For a set of decisions about health and personal finance, we used quantitative (e.g., decision accuracy) and qualitative (e.g., free-text descriptions of decision processes) methods to capture decision quality and how people used the provided information. We found that participants given causal information focused on different aspects than did those who did not receive causal information and that reasoning linked to better decisions with no information was associated with worse decisions with causal information. Furthermore, people brought in many aspects of their existing knowledge and preferences, going beyond the conclusions licensed by the provided information. Our findings provide new insights into why decision quality differs systematically between familiar and novel scenarios and suggest directions for future work guiding everyday choices.

## 1 Introduction

We use causal reasoning when deciding to take medication (to alleviate symptoms), save money for retirement (to ensure a comfortable old age), or try a diet (to modify our body weight). We select actions because we believe they can bring about desired effects (Kleinberg, 2013). Our beliefs may be correct, such as recognizing a medication's ineffectiveness for ourselves, or faulty, such as believing “lucky” socks determine the outcome of a game. Beliefs inform our everyday decisions, influence whether we seek out new information, and affect how we use it.

Yet little is known about how beliefs influence our receptivity to new information and ability to use it to make decisions. Information seeking has been extensively studied for health (Anker et al., 2011), particularly regarding where people obtain health information (Korshakova et al., 2022). People often have incorrect health beliefs, such as thinking preventable illnesses cannot be prevented (Smith et al., 1999) or believing in conspiracy theories about health, as almost half of Americans do (Oliver and Wood, 2014). Such misconceptions are not limited to health. Lay beliefs also influence how we use new information. (Kleinberg et al. 2023) found that, even when incorrect, beliefs affected the perceived reasonableness of options, suggesting that evidence may not correct entrenched beliefs. Thus, understanding how beliefs interact with causal information is important.

In causal cognition, actions are a form of information gathering. After we take an action, we see its result and may behave differently next time. The types and roles of interventions chosen have been examined in the context of how people learn causal models (Bramley et al., 2017; Coenen et al., 2015), but in artificial domains where people cannot use prior knowledge, such as learning how a made-up machine works. We face a different problem in everyday choices as experience can influence perceptions of new information. For example, omitting information people expected to see in a causal model reduced their trust in it (Kleinberg et al., 2022). This finding is only possible because the questions were about topics such as how gray hair develops. In a novel domain in which participants are dependent on the information provided by researchers, they do not know what other information could be included and might be missing. This highlights a core difference: In familiar domains, we bring prior beliefs that may be hard to change.

Research testing decision-making with causal models in familiar domains, such as managing health and finances, revealed a puzzle: Causal information can lead to worse choices on familiar topics while aiding people in novel domains (Zheng et al., 2020). Before this, much work on causal cognition found that people are adept at learning and using causal models (Bramley et al., 2017; Lagnado and Sloman, 2004, 2006; Rottman, 2017) but focused on novel domains in which participants relied on the information provided and could not use their prior experiences, preferences, or knowledge. Recent work on domains in which people have varying experience (e.g., life choices such as buying a house) found that narrowly targeted information can improve decisions, while extra information led to worse choices (Kleinberg and Marsh, 2023). Highlighting the relevant paths within a complex model led to similar results as when only that information was presented, suggesting that people may have difficulty determining when which parts of a model are most relevant.

Engaging with a causal model may also lead to different types of reasoning. (Liefgreen and Lagnado 2023) found that participants who drew and updated causal models of legal evidence after hearing each side cited different reasons for judgments than participants who were asked only to describe the evidence. In particular, participants who drew causal models preferred simpler explanations. This work focused on the legal domain, so whether, in general, people use different types of reasoning depending on whether they use causal models is an open question.

Causal information is pervasive and should lead to better choices as it provides effective strategies for intervention. Prior work, however, suggests that this may not be true in familiar domains, but as it only examined decision accuracy, it could not fully answer this question. We addressed this open question in an exploratory study using a mixed-methods approach, eliciting qualitative information about how people make decisions with and without causal models in two domains (finance and health), along with quantitative information on decision accuracy. We examined (1) whether different types of reasoning are used with causal information compared to when people make decisions using their existing knowledge, (2) whether specific types of reasoning are associated with the correctness of decisions, and (3) whether consistency between prior beliefs and causal information was associated with decision accuracy.

## 2 Methods

### 2.1 Participants

We recruited 337 U.S. residents aged 18–65 through Prolific.

### 2.2 Materials

We tested decision-making in everyday scenarios (health and finance) in which people are expected to bring existing knowledge and beliefs. We used two scenarios (managing body weight and saving for retirement) previously shown to yield worse accuracy with causal information than without (Zheng et al., 2020). We additionally used two questions about Type 2 diabetes (T2D): one in which a person aims to reduce their risk of T2D (prevention) and another in which a person must identify and treat an instance of low blood glucose (explanation). The prevention question was also used by (Kleinberg and Marsh 2020). The explanation question is intended to be challenging as participants must determine from symptoms whether the person's blood sugar is high or low and then select an appropriate intervention. The T2D questions follow:

#### 2.2.1 Explanation question

*Amy has Type 2 diabetes, which means her body does not produce enough insulin to keep her blood sugar in a healthy range. To help manage her blood sugar, she exercises regularly*.

*This morning she went for a run that was a bit longer than usual. Once she got to work she had a big breakfast*.

*Now she's feeling hot and shaky, and her vision is blurry*.

*If you were advising Amy on how to reduce her symptoms, what would you suggest she do now?*

A. Walk slowly until she feels better.
B. Give herself an injection of insulin.
C. Drink a can of regular soda.
D. Eat some potato chips.

The correct answer for this question is C because Amy has low blood sugar, and this is the only option that can quickly raise her blood sugar.

#### 2.2.2 Prevention question

*Robert's mother has Type 2 diabetes. His doctor recently told him he is at risk as well. He stopped eating fast food in the hopes of reducing his weight, but his blood pressure remains high*.

*Robert's town is very hot in the summer and very snowy in the winter, so he tends to drive even short distances*.

*After seeing his mother's diabetes complications, he is concerned about his risk but doesn't know what he can do about it*.

*What is the BEST suggestion you can give Robert to reduce his risk of diabetes?*

A. Spend less time with his mother.
B. Don't do anything, risk can't be reduced.
C. Take medication for his blood pressure.
D. Walk instead of driving twice a month.

The correct answer is C because Robert's high blood pressure puts him at risk of diabetes.

We developed causal information (diagram and text matched for content) designed to aid participants in selecting the right answer. Supplementary Figure S1 shows the diagram for the prevention question.

### 2.3 Procedure

**Figure 1:**
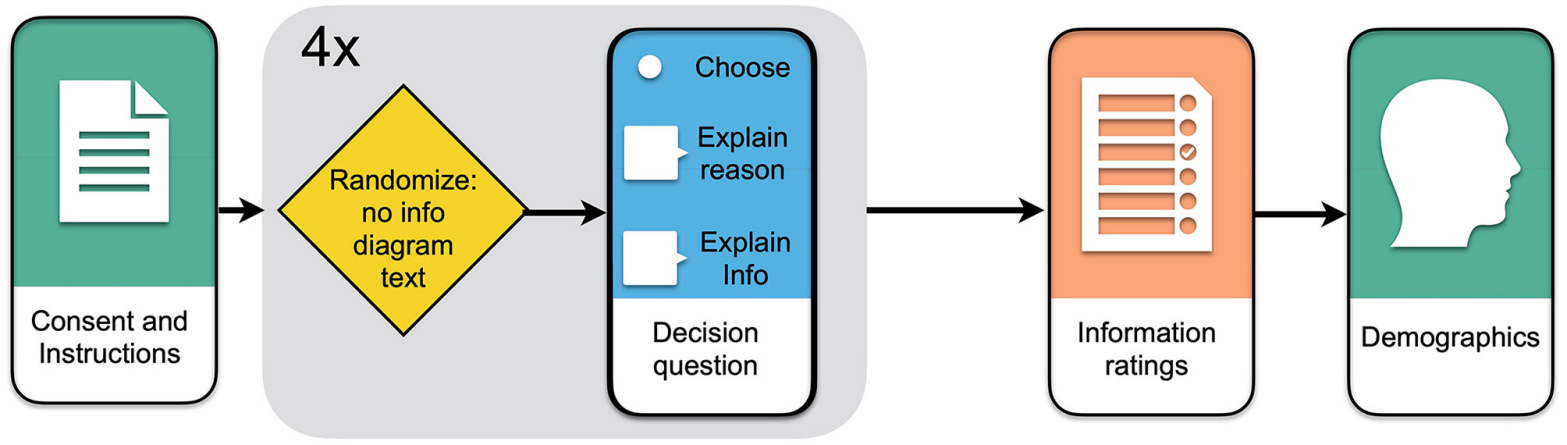
Overview of study process. Participants began by consenting to the study and reading instructions on the procedure. Participants were randomized by question to the three information conditions and completed decision-making and free-text responses for each. After the decision questions participants completed ratings on the information provided. Finally, participants provided demographic information.

Figure 1 shows an overview of the procedure. After consenting, participants were instructed that “some questions may have figures or text that tell you more about the problem” and instructed about the meaning of causal diagrams. Each participant saw the four decision-making questions in a randomized order, with a randomized information condition for each question (no information, diagram, and text). On the same page as each decision-making question, participants were provided two text boxes and prompts to (1) explain their reasoning in as much detail as possible (reasoning) and (2) explain what information they used to make their choice (information). Following this, for each question in which participants saw causal information (diagram or text), they rated the following statements about its utility on a scale of 0 (*strongly disagree*) to 7 (*strongly agree*):

- The diagram was easy to understand (understandable).
- The diagram was informative and increased my understanding about the topic (informative).
- The diagram increased my confidence in my answer to the question (confidence).
- I strongly believe the relationships shown in this diagram (believable).
- The diagram closely represented my own ideas and beliefs about the topic (compatible).

Below these statements, participants were provided two text boxes and prompts to explain (1) how their ideas and beliefs on this topic differed or were similar to the information presented (similarity) and (2) any changes they would make to the diagram and/or the information in the diagram (changes). The statements were modified to refer to text rather than diagrams for text condition questions. Finally, participants completed a demographic questionnaire.

### 2.4 Analysis approach

We had two types of data: quantitative (decision accuracy and numerical ratings of statements) and qualitative (free-text responses about decision process and match between information provided and beliefs). The free-text responses about participants' decision process (reasoning and information) were combined for analysis due to the similarity of responses. We refer to the combined category as “reasoning” and hand-coded items through an iterative process (Saldaña, 2021). The coding scheme was developed by two coders, after which items were coded by the second author. We identified frequent phrases and themes and combined related codes as themes emerged (e.g., combining “common sense” and “reading articles” into “prior knowledge”). Some key words for a theme differed by topic, such as specific descriptions of side effects. Themes were coded as present or absent for each participant for each question. To analyze the relationship between themes and accuracy, we used the generalized linear mixed-effects models in *glmer* with the outcome being correctness, with a random intercept for each participant, using the themes as fixed effects and including interactions with information condition.

In coding text feedback about the information provided, we aimed to identify to what extent participants felt the information matched their beliefs or whether another type of judgment was made. We again identified keywords and classified the main themes. Examining consistency with beliefs, the final themes were match (information is fully consistent with beliefs), partial match (information overlapped but did not completely capture beliefs), and no match (information was incongruent with beliefs). Participants also expressed judgment about the text or diagram's value, so we coded the following themes: overcomplicated (information is too complicated), oversimplified (information is too simple), and unclear (information was not clear). Finally, two further themes emerged with participants sharing the extent to which they relied on the information provided: “Experience” indicated that participants ignored the information given and answered based solely on their own personal experience, while “info from question” indicated the opposite—they based their answer solely on the information provided because they did not have any strong beliefs about the topic going into the experiment. We checked for multicollinearity using the variance inflation factor (VIF). VIF values for all themes were between 1 and 1.1, indicating low to no multicollinearity.

## 3 Results

### 3.1 Decision process

#### 3.1.1 Participants combined prior knowledge, preferences, and information

Participants were *M* = 35.08 years (*SD* = 11.74), and 74.5% were women; 24.9%, men; and 0.6%, other. Participants mentioned many factors beyond the information provided, including perceived side effects, difficulty or ease of implementation (feasibility), and the participant's own preferences. Following is a sample of reasons given by participants for picking specific choices:

- “Waking *[sic]* will improve her mood and is likely to improve her weight also. If she gave up her social time then she would be less likely to walk because she would be unhappy” (side effect, assumptions).
- “She has to walk to her classes anyway which is probably 30 min of activity combined” (assumptions).
- “Frankly, I feel that we're overmedicating people, so I just went with good old exercise as the best option” (preferences).
- “I looked at the options and chose the one i would pick as I myself am type 1 diabetic so know that when i feel similar after exercise, that is what i would do” (preferences).
- “Maintaining a healthy diet is the hardest thing to do as its [*sic*] an everyday activity, adding exercise 3 times a week is easier” (feasibility).
- “Maintaining a healthy diet is one of the easier options” (feasibility).
- “I thought that it would be a higher risk to have assets in a wide range of products which change prices regularly, as I would have assumed that it would be harder to keep track of all the investments and that it would be more volatile if the prices are changing more often” (feasibility).

Participants in all conditions referenced beliefs and preferences beyond the information provided. Supplementary Table S1 shows that while the percentage of mentions of information from the question was stable across topics, other reasoning varied, with feasibility often mentioned when considering changes to diet (31%) or medication (36%) and side effects being mentioned for financial choices (81%) and managing body weight (32%). Notably, the same theme can lead to different choices, as in the previous example where two participants considered the ease of an action but came to different conclusions about which factor is easiest to modify (one stating that diet is difficult to modify and another saying it is easier). Given the number of personal factors mentioned, we further examined how frequently participants referred to themselves, finding 15% of responses involved self-mentions without mentions of experience.

#### 3.1.2 Accuracy varied by reasoning type

We next examined whether different reasoning was mentioned in each information condition and whether type of reasoning was associated with differences in decision accuracy. We replicated prior results from Zheng et al.'s (2020) study, finding that accuracy for text (53.5%) and diagram (50.1%) did not differ significantly (*p* = 0.31), while accuracy for no information (61.6%) was significantly higher than both (*p* = 0.01 and *p* < 0.001, respectively). Given the demographic imbalance, we reran our models with age and gender as covariates, although neither was significant (see Supplementary material).

**Figure 2:**
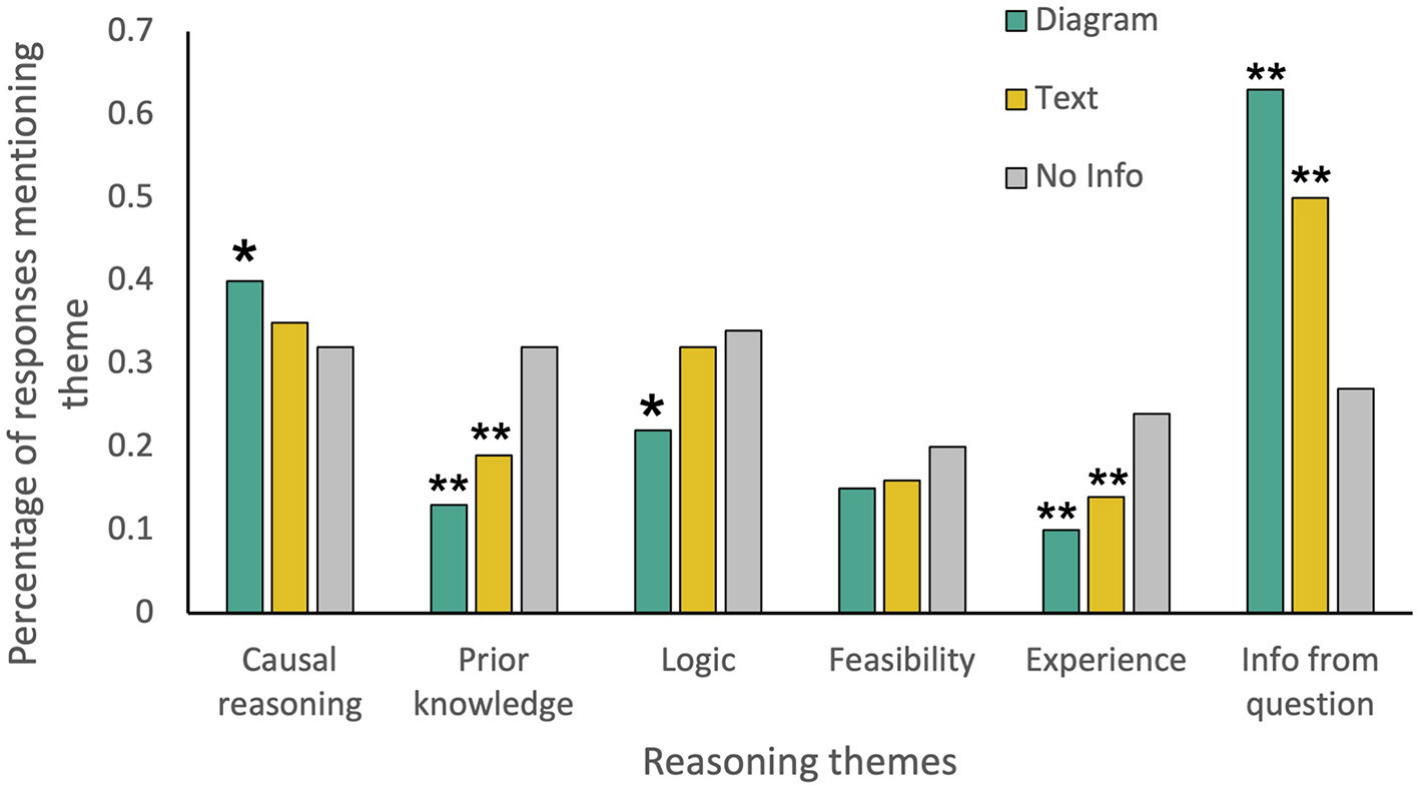
Percentage of responses mentioning each theme by condition, collapsed across topics. Significant differences between information condition and no info with N−1 chi-square are marked. *p < 0.05. **p < 0.001.

**Figure 3:**
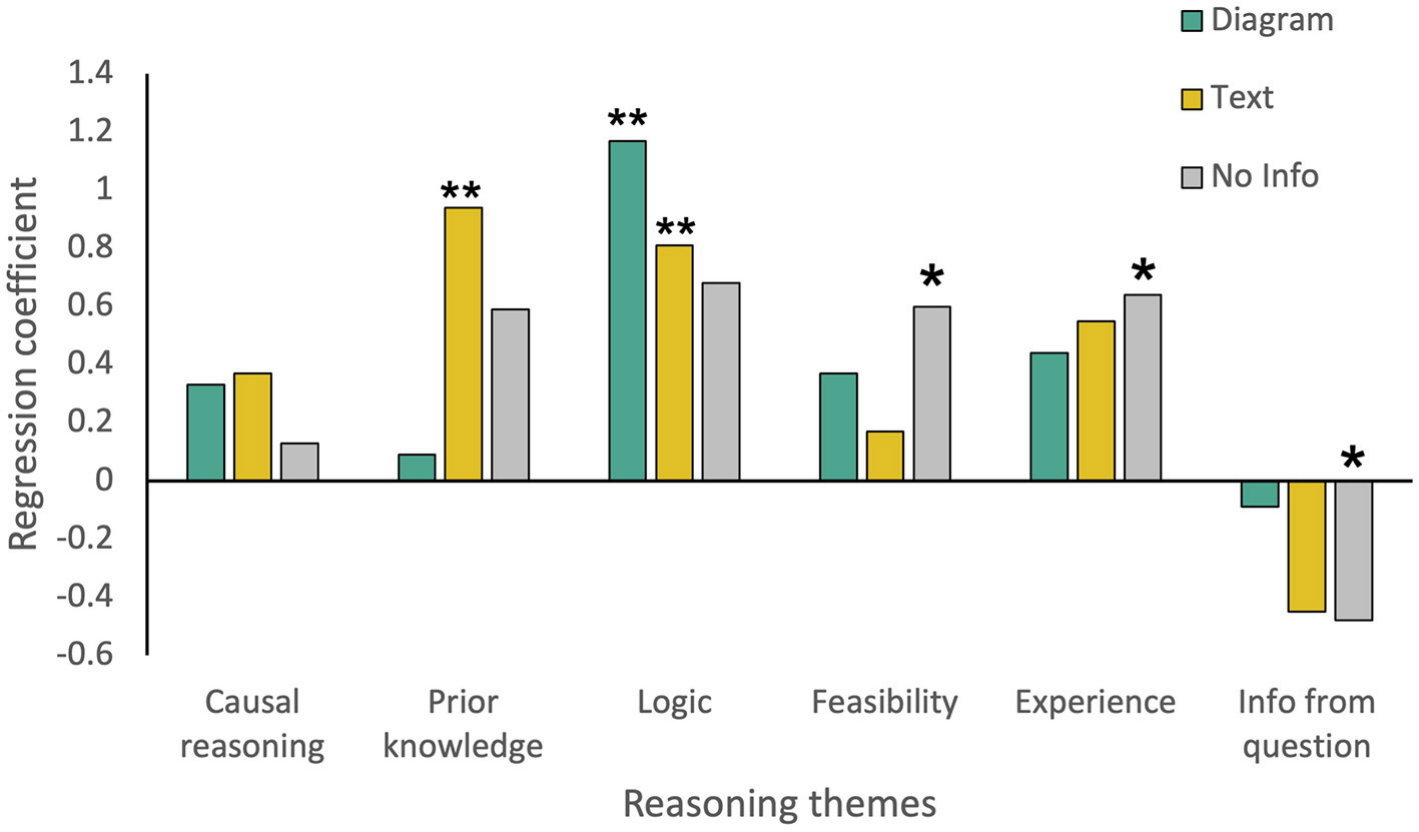
Regression coefficients for model predicting accuracy as a function of reasoning theme for. Coefficients' significance is marked. *p < 0.05. **p < 0.001.

As shown in Figure 2, participants used different types of reasoning in the different information conditions, with prior knowledge and experience with no info being used significantly more frequently compared to the diagram and text and info from the question being used more in the diagram and text conditions. Causal reasoning was used significantly more in the diagram condition compared to the no-info condition (*X*^2^ = 4.72, *p* = 0.03). We examined whether reasoning type predicted accuracy in each information condition using logistic regression, with one model predicting accuracy in each condition as a function of mentions of each theme (Figure 3, Supplementary Table S3). The types of reasoning that predicted high accuracy with no information (prior knowledge, feasibility, and experience) did not predict accuracy with causal diagrams. Notably, info from the question had a significant negative relationship with accuracy for text and no information. Logic had a positive relationship with accuracy in all conditions, and prior knowledge was significant in the text condition.

### 3.2 Causal information interpretation

Our first analysis focused on the decision-making process. We now examine how people related the information shown to their existing beliefs and whether this was associated with decision accuracy.

#### 3.2.1 Participants used prior knowledge to interpret information

The questions were not answered for no info, so we combined the text and diagram conditions for the qualitative analysis. As with the decision-making questions, participants relied on their background knowledge in interpreting the information provided. Representative responses about the causal diagrams were:

- “it does provide information but like other charts the information isn't complete. There has to be a clear picture of his risk appetite and how much would he want to reduce the risks by and what's the cost involved in reducing the risk and how it impacts the return on investment” (oversimplified).
- “I think the information is accurate, but a little oversimplified. There are many other factors that would contribute to drinking alcohol. Also, abstinence from something is not always a realistic goal, but the diagram doesn't account for something healthier, like occasional drinking. I also tend to believe diet has a greater cause on weight than exercise” (oversimplified).
- “My ideas and beliefs were fairly well represented in the diagram, but not completely. For example, whilst I agree with the theory that social pressure whilst at college might increase the likelihood of consuming alcohol, we are ultimately in control of our own minds and have the right to decline. So it's still perfectly possible to fall victim to social pressures and have a perfectly healthy weight” (partial match).

Supplementary Table S2 shows the percentages of participants who mentioned each theme for each question. For the body weight, finance, and prevention questions, many participants mentioned consistency between their beliefs and the information provided (67%, 50%, and 55% match, respectively). However, this code's prevalence was lower for the explanation question (37%), and 33% of participants said that they relied on the information provided because they did not know much about the topic.

#### 3.2.2 Accuracy varied by information match

**Figure 4:**
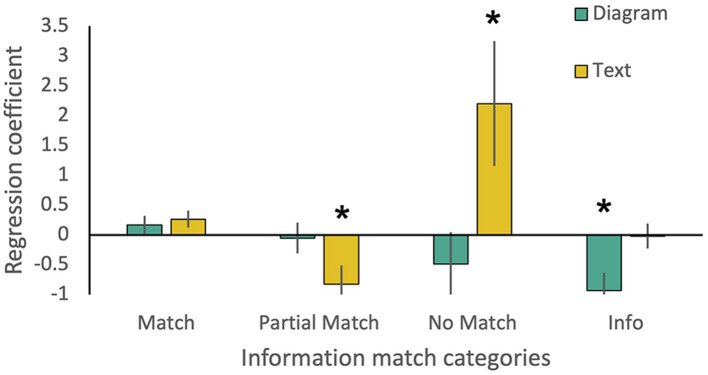
Regression coefficients for model predicting accuracy as a function of information match for text and diagram conditions. Coefficients with *p* < 0.05 are marked with an asterisk.

We examined the connection between information match themes and decision accuracy using a regression with degree of match (match, partial match, no match, and info) to predict correctness. As shown in Figure 4, for diagram condition, the only significant predictor of accuracy was info (where participants used only the information provided), while for the text condition, a partial match was significantly negatively associated with accuracy, and no match was a positive predictor (see Supplementary Tables S4, S5 for the full model). Participants relying fully on their own knowledge or fully on the information provided performed best while facing difficulties when reconciling information sources (i.e., with partial match). Supplementary Table S6 shows participant ratings for each information type.

## 4 Discussion

Prior work revealed a conflict: People are adept at learning causal models and using them for decision-making in novel domains (Rottman, 2017), yet causal models can lead to worse choices in familiar domains (Zheng et al., 2020). Our results help reconcile why causal models aid decisions in novel domains but hinder them in familiar ones by showing that (1) people use different types of reasoning when making decisions with causal information, (2) different reasoning types predict accuracy with and without causal information, and (3) perceived conflict between a causal model and beliefs may yield worse choices. These results have implications for understanding cognition and decision-making and methodology.

(Zheng et al. 2020) first showed that causal models could yield worse decisions compared to no information. (Kleinberg and Marsh 2023) found that providing subsets of a model (i.e., only the relevant causal paths) improved accuracy, while including extra information led to worse results. Our results offer a potential mechanism and explanation why this effect is absent in novel contexts: In familiar domains, people do not use only the information provided. In both information conditions (diagram and text), participants incorporated their prior knowledge, their beliefs about feasibility of actions, and their own experiences, while in novel setups, participants cannot bring in experience and knowledge. While participants were more likely to reason logically when given no information compared to causal diagrams, logic significantly predicted accuracy in all three conditions. Reliance on outside information has been examined for causal attribution (determining who and/or what caused or is responsible for an event). Faced with a sparsely described vignette, people introduce additional assumptions and inferences to assign causality and responsibility (Alicke, 2000; Hilton, 2017; Kirfel and Lagnado, 2021). Taken together, this suggests a need for future work to better understand how causal reasoning differs in familiar domains in which people are able to add in details beyond the vignette.

We further found that belief conflict may contribute to how well people use causal information. A partial match between beliefs and information was negatively associated with accuracy in the text condition and may be especially challenging due to increased cognitive load or dissonance as people attempt to reconcile new information with conflicting beliefs (Yang et al., 2024; Harmon-Jones et al., 2009). With a full match, people do not need to resolve differences between mental and causal models, while with no match, people may ignore the information and use prior knowledge. In contrast, partial overlap may increase difficulty in making decisions. Conversely, when participants reported using only the information provided, the caveat is that with realistic causal models, some degree of interpretation is still required (e.g., translating exercise recommendations into specific activities and frequencies). Prior work has shown that causal evidence can be persuasive in changing beliefs (Slusher and Anderson, 1996; Hornikx, 2005), yet many other works show how difficult changing beliefs is (Ecker and Antonio, 2021; Jelalian and Miller, 1984) and that new information does not change our rating of decision options (Kleinberg et al., 2023). However, we did not assess accuracy of participants' prior knowledge. Potentially, the individuals who most need information may ignore it or struggle to reconcile it with their own beliefs. More work is needed to understand how to help people assimilate new information into their decision-making.

Our findings have significant methodological implications for studying causal reasoning. Prior work often used novel setups (e.g., blicket detectors), creating a controlled setting to fully manipulate a participant's information. While these studies provide insight into cognition, such as how people use observation and intervention to learn causal models (Steyvers et al., 2003), our study suggests that results may not be representative of those in familiar domains. For example, the degree to which participants relied on prior knowledge in our study and how experience influenced their choices (e.g., considerations about which interventions are easier) could not be predicted by studies with novel stimuli. Thus, examining the role of knowledge and experience in how people learn about and use causal information more fully is needed.

Finally, our research has practical implications for how to present causal information to maximize its benefit to a decision-maker. While (Kleinberg and Marsh 2023) showed that the simplest information leads to the best decisions, we now see that the match between a decision-maker's knowledge and the information presented also plays a role. When giving health advice to patients, understanding the relationship between their beliefs and information in decision aids is crucial. A complex diagram that does not capture their beliefs could do more harm than no information, whereas an overly simplistic diagram could be overridden by an individual's prior and possibly faulty knowledge.

Our results have some limitations. First, because decisions were hypothetical, we cannot say whether the same reasoning would be used for real-world decisions. However, because the decisions were realistic and not high stakes, we expect similar patterns in real-world contexts. Second, we did not assess participants' knowledge, so we could not distinguish between perceived vs. actual knowledge in our models. We expect that the key factor is perceived knowledge because people may be incorrect in their self-assessment. We are currently testing this in other work. Furthermore, our study was exploratory in nature, as we aimed to discover themes from the qualitative data. Now that we have identified key features related to decisions with causal information, future work can now test these hypotheses with preregistered studies (with predefined codes and criteria for assigning them) to replicate results. Finally, we studied two common areas of decision-making (health and finance) to keep the length of our study manageable for participants, but it is possible that results may differ for other domains. Future work is needed to examine the role of domain.

We use causal knowledge to navigate the world, yet little is known about how our prior knowledge and experiences shape how we interpret new information. Through qualitative and quantitative analysis of decision-making in everyday domains, we found that people engage in different reasoning with causal information compared to using existing knowledge, that different reasoning processes are associated with decision accuracy in each setting, and, finally, that the degree of concordance between prior beliefs and causal information is related to decision accuracy. Our work highlights the need to study decision-making in familiar domains and examine the role of prior causal beliefs in decision-making. Our findings may ultimately be used to improve communications for the lay public for decision-making.

## Data Availability

The raw data supporting the conclusions of this article will be made available by the authors upon request.
