# Supplementary material for: Causal information changes how we reason: a mixed-methods analysis of decision-making with causal information
Source: Front Cognit. 2025 Aug 11;4:1608842. doi: 10.3389/fcogn.2025.1608842 (PMC13271127; doi:10.3389/fcogn.2025.1608842)
Supplement: Supplementary file 1 [file Table_1.docx]

**Causal Information Changes How We Reason: A Mixed Methods Analysis of Decision-Making with Causal Information**

**Samantha Kleinberg^1^, Cristina Leone^2^, Alice Liefgreen^2^, David A. Lagnado^2^**

^1^Computer Science Department, Stevens Institute of Technology, Hoboken, NJ, USA

^2^Experimental Psychology, Division of Psychology & Language Sciences, University College London, London, United Kingdom

**Supplementary Materials**

**Additional Results**

Given the demographics of our participants, we additionally included age and sex as covariates to test whether either was associated with decision accuracy. We again used a linear mixed model with information condition, domain, and their interaction, along with gender (binary, excluding 2 individuals identifying as “other”), and age (continuous). Coefficients for age (p =.56) nor gender (.52) were not significant in the model.

**Tables and Figures**

*Table S1. Percentage of participants mentioning each reasoning theme by question, collapsed across all information conditions.*

| Theme | Description | Bodyweight | Finance | BP/Prevention | Hypoglycemia/  Explanation |
| --- | --- | --- | --- | --- | --- |
| feasibility | Being easier or more practical | 0.31 | 0.0 | 0.36 | 0.09 |
| logic | Using logic or deduction, process of elimination | 0.38 | 0.44 | 0.13 | 0.21 |
| experience | Using personal experience | 0.26 | 0.15 | 0.10 | 0.18 |
| info from question | Mention of diagram, information provided | 0.46 | 0.40 | 0.47 | 0.50 |
| prior knowledge | Common sense/ knowledge, prior reading | 0.24 | 0.27 | 0.13 | 0.20 |
| causal reasoning | Using causal language | 0.44 | 0.21 | 0.31 | 0.39 |
| avoiding side effects | Mention of negative impacts of a choice | 0.32 | 0.81 | 0.14 | 0.07 |
| benefits | Mention of positive impacts of a choice | 0.00 | 0.00 | 0.12 | 0.00 |

*Table S2. Percentage of participants mentioning each similarity theme by question, collapsed across text and diagram information conditions.*

| Theme | Description | Bodyweight | Finance | BP/Prevention | Hypoglycemia/  Explanation |
| --- | --- | --- | --- | --- | --- |
| match | information fully consistent w/ beliefs | 0.67 | 0.50 | 0.55 | 0.37 |
| partial match | information partly consistent w/beliefs | 0.16 | 0.03 | 0.23 | 0.07 |
| no match | information does not match beliefs | 0.02 | 0.05 | 0.02 | 0.03 |
| overcomplicated | information too complex | 0.01 | 0.00 | 0.00 | 0.01 |
| oversimplified | information too simple | 0.07 | 0.00 | 0.05 | 0.00 |
| unclear | information unclear | 0.00 | 0.12 | 0.03 | 0.15 |
| experience | relied fully on own experience | 0.04 | 0.00 | 0.03 | 0.05 |
| information | relied fully on provided information | 0.01 | 0.23 | 0.10 | 0.33 |

*Table S3. Reasoning type as a predictor of decision accuracy, by condition.*

|  | Diagram | | Text | | | No info | | |
| --- | --- | --- | --- | --- | --- | --- | --- | --- |
|  | Estimate | P-value | Estimate | P-value | Estimate | | P-value |  |
| Causal reasoning | 0.33 | .09 | 0.37 | .08 | 0.13 | | .54 |  |
| Prior knowledge | 0.09 | .74 | 0.94 | <.001*** | 0.59 | | .01* |  |
| Logic | 1.17 | <.001*** | 0.81 | <.001*** | 0.68 | | .003** |  |
| Feasibility | 0.37 | .21 | 0.17 | .52 | 0.60 | | .02* |  |
| Experience | 0.44 | .19 | 0.55 | .06 | 0.64 | | .01* |  |
| Info from question | -0.09 | .65 | -0.45 | .02* | -0.48 | | .03* |  |

Table S4. Accuracy as a function of match between a participant’s beliefs and information provided for diagram condition.

| Diagram | Regression Coefficient | Standard Error | Z value | P value |
| --- | --- | --- | --- | --- |
| Match | 0.17 | 0.149 | 1.146 | .25 |
| Partial Match | -0.053 | 0.255 | -0.21 | .83 |
| No Match | -0.49 | 0.54 | -0.918 | .36 |
| Info | -0.933 | 0.30226 | -3.088 | **.002**** |

*Table S5. Accuracy as a function of match between a participant’s beliefs and information provided for text condition.*

| Text | Regression Coefficient | Standard Error | Z value | P value |
| --- | --- | --- | --- | --- |
| Match | 0.26442 | 0.14 | 1.837 | .066 |
| Partial Match | -0.82753 | 0.31638 | -2.616 | **.009**** |
| No Match | 2.20 | 1.04548 | 2.110 | **.034*** |
| Info | -0.02 | 0.20776 | -0.139 | .89 |

Table S6. Participant ratings of each item by question and information type.

|  | | Understandable | Informative | Confidence | Believable | Compatible |
| --- | --- | --- | --- | --- | --- | --- |
| Bodyweight | Text | 6.1 | 5.3 | 5.4 | 5.4 | 5.5 |
|  | Diagram | 5.7 | 5.1 | 5.0 | 5.3 | 5.3 |
| Finance | Text | 5.1 | 4.9 | 4.7 | 4.8 | 4.6 |
|  | Diagram | 4.8 | 4.3 | 4.3 | 4.5 | 4.2 |
| BP/Prevention | Text | 5.9 | 5.3 | 5.3 | 5.3 | 5.3 |
|  | Diagram | 5.0 | 4.9 | 4.9 | 5.1 | 5.0 |
| Hypoglycemia/Explanation | Text | 5.2 | 5.2 | 4.9 | 5.0 | 4.8 |
|  | Diagram | 4.3 | 4.4 | 4.2 | 4.5 | 4.0 |

Table S7. Confidence intervals and odds ratios for model of accuracy as a function of reasoning type.

| Reasoning Theme | Odds ratio estimate | 95% C.I. |
| --- | --- | --- |
| Info from question | 0.692 | (0.55-0.87) |
| Feasibility | 1.467 | (1.08-2.01) |
| Logic | 2.321 | (1.80-3.01) |
| Experience | 1.791 | (1.30-2.48) |
| Prior Knowledge | 1.829 | (1.37-2.45) |
| Causal | 1.309 | (1.04-1.66) |

**
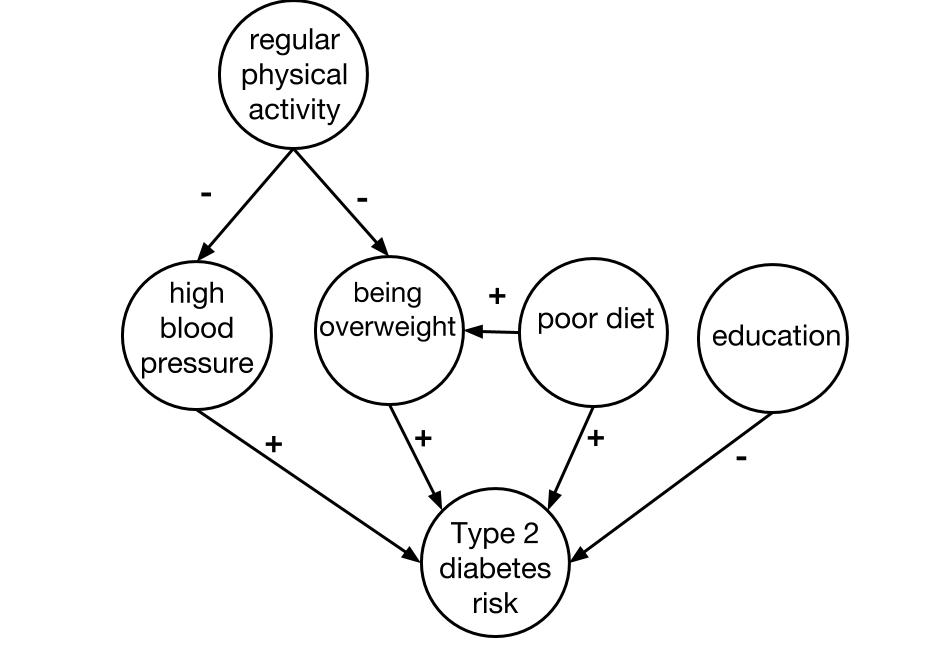
**

*Figure S1. Example causal diagram corresponding to question about reducing T2D risk.*
